# Insights into brain metastasis in patients with *ALK*+ lung cancer: is the brain truly a sanctuary?

**DOI:** 10.1007/s10555-015-9592-y

**Published:** 2015-09-05

**Authors:** Gouji Toyokawa, Takashi Seto, Mitsuhiro Takenoyama, Yukito Ichinose

**Affiliations:** Department of Thoracic Oncology, National Kyushu Cancer Center, 3-1-1 Notame, Minami-ku, Fukuoka, 811-1395 Japan

**Keywords:** Non-small cell lung cancer, Anaplastic lymphoma kinase (ALK), Brain metastasis, ALK inhibitors

## Abstract

*Anaplastic lymphoma kinase* (*ALK*) has been identified to exert a potent transforming activity through its rearrangement in non-small cell lung cancer (NSCLC), and patients (pts) with *ALK* rearrangement can be treated more successfully with ALK inhibitors, such as crizotinib, alectinib, and ceritinib, than with chemotherapy. Despite the excellent efficacy of ALK inhibitors, resistance to these drugs is inevitably encountered in most *ALK*-rearranged pts. Cases of resistance are subtyped into three groups, i.e., systemic, oligo, and central nervous system (CNS) types, with the CNS being used to be considered a *sanctuary*. With regard to the management of CNS lesions in pts with *ALK*+ NSCLC, a growing body of evidence has gradually demonstrated the intracranial (IC) efficacy of ALK inhibitor (ALKi) in *ALK*+ NSCLC pts with brain metastases (BMs). Although the efficacy of crizotinib for the CNS lesions remains controversial, a recent retrospective investigation of *ALK*+ pts with BM enrolled in PROFILE 1005 and PROFILE 1007 demonstrated that crizotinib is associated with a high disease control rate for BM. However, BM comprises the most common site of progressive disease in pts with or without baseline BMs, which is a serious problem for crizotinib. Furthermore, alectinib can be used to achieve strong and long-lasting inhibitory effects on BM. In addition to alectinib, the IC efficacy of other next-generation ALK inhibitors, such as ceritinib, AP26113 and PF-06463922, has been demonstrated. In this article, we review the latest evidence regarding the BM and IC efficacy of ALK inhibitors in pts with *ALK*+ NSCLC.

## Introduction

Rearrangement in the *anaplastic lymphoma kinase* (*ALK*) gene is one of the potent oncogenic drivers in patients (pts) with non-small cell lung cancer (NSCLC) and occurs in approximately 5 % of cases [1, 2]. Tyrosine kinase inhibitors (TKIs) against ALK, such as crizotinib, alectinib, and ceritinib, have recently been developed and shown to exert a potent antitumor activity in pts with *ALK*-rearranged NSCLC [3–9]. With regard to crizotinib, which has been originally developed as a c-Met inhibitor, the response rate (RR) and progression-free survival (PFS) of crizotinib in first- and second-line settings for advanced *ALK*+ NSCLC are 74 and 65 % and 10.9 and 7.7 months, respectively, which are significantly better than those achieved with standard chemotherapy [5, 6]. Alectinib, a next-generation ALK inhibitor (ALKi), is also known to exhibit a high antitumor activity for pts with *ALK*+ NSCLC, i.e., RR and PFS of 93.5 % and 27.7 months, respectively [7]. Critically, alectinib is effective for *ALK*+ NSCLC pts with a history of prior crizotinib treatment [8] and ceritinib has been shown to demonstrate high efficacy for pts with *ALK*+ NSCLC treated with or without prior therapy with crizotinib [9].

Metastasis to the central nervous system (CNS) is a difficult concern to be resolved in many types of cancer as a result of inadequate penetration of antitumor agents, difficult accessibility, and neurological symptoms leading to a decreased performance status, and lung cancer is the leading cancer to metastasize to the brain. According to the SEER database, lung cancer occurred in 19.9 % of 16,210 pts, followed by melanoma at 6.9 % [10, 11]. In pts with advanced NSCLC, approximately 10–20 % of cases are considered to be accompanied by CNS metastases prior to first-line treatment [12, 13]. With regard to the incidence of brain metastasis (BM) in pts with *ALK*+ NSCLC who are naïve to ALKi, several retrospective studies have reported rates ranging from approximately 20 to 30 %, which is comparable to that observed in *epidermal growth factor receptor* (*EGFR*)-mutated NSCLC pts [14, 15]. In large trials examining ALKi, the frequency of BM in ALKi-naïve pts has been reported to range from approximately 25 to 40 %, which is high in pts with a history of chemotherapy [5–7, 16]. Furthermore, *ALK*-rearranged NSCLC pts exhibiting a history of prior ALKi treatment are reported to harbor a high incidence of CNS metastases, i.e., from approximately 45 to 70 %, which suggests that BM is the most common pattern of failure of ALKi [8, 9, 17–21]. Therefore, it is crucial to properly treat CNS lesions as well as systemic lesions.

With regard to the efficacy of ALKi for BM in *ALK*+ NSCLC pts, the efficacy of crizotinib was analyzed in retrospective analyses of the PROFILE 1005 and PROFILE 1007 trials [22]. The disease control rate (DCR) and RR in previously untreated and treated BM cases were 56 and 62 %, and 18 and 33 %, respectively. In addition, several reports have indicated that alectinib exhibits excellent antitumor efficacy for CNS metastases in *ALK*+ NSCLC pts with or without prior ALKi treatment, including crizotinib and ceritinib [7, 8, 19, 20, 23–25]. According to the results of AF-002JG, the intracranial (IC) objective RR was intriguingly 52 %, with a complete response rate of 29 % [8]. In concordance with the clinical efficacy observed in *ALK*+ pts, the remarkable efficacy of alectinib has been demonstrated in experimental models of IC metastases [26, 27]. The high efficacy of alectinib for *ALK*+ CNS metastases is considered to be due to its high penetration to the brain as well as poor efflux from the brain, owing to experimental results demonstrating that alectinib might not be transported by *P*-glycoprotein (*P*-gp) efflux protein, a key protein for blood-brain barrier (BBB) penetration [26]. In addition to alectinib, ceritinib has been shown to be effective for BM, and novel compounds, such as AP26113 and PF-06463922, have also been demonstrated to be effective for CNS lesions [16–18, 21, 28].

Therefore, a growing body of evidence has been accumulated for BM in pts with *ALK*+ NSCLC. In this review article, we review up-to-date evidence on recent clinical and basic results regarding BM and discuss the optimal management of BM in pts with *ALK*-positive NSCLC.

## Incidence of IC metastases in pts with *ALK*+ NSCLC

The brain is a devastating metastatic site of malignancies, including lung cancer, as metastasis to the brain might result in neurological dysfunction and cognitive impairment, which can lead to an impaired quality of life. The presence of IC metastases is known to be associated with a poor prognosis. According to the report by Paul and colleagues, the median survival time of pts with BM is 7.0 months from the start of treatment of BM and, in particular, pts at high risk, as defined based on various determinants, including the number of BM lesions, achieve a survival as short as 3.0 months [29].

ALK is normally expressed in the CNS, small intestine, and testes and is considered to play a role in the development of the nervous system; however, its involvement in IC metastasis and its significance in normal organs have yet to be elucidated [30]. With regard to the baseline frequency of BM in pts with *ALK*+ NSCLC at the time of diagnosis of the disease, two retrospective studies showed a frequency of approximately 25 %, which is comparable to that observed in other subsets of NSCLC driven by *EGFR* and *Kirsten rat sarcoma viral oncogene homolog* (*KRAS*) [14, 15]. This incidence is consistent with that reported in the PROFILE 1014 trial, which compared crizotinib with chemotherapy in pts with *ALK*-rearranged NSCLC as a first-line therapy; i.e., the frequency of BM in both arms was 26 and 27 %, respectively [6].

With regard to the incidence of CNS metastases in ALKi-naïve *ALK*+ NSCLC pts previously treated with chemotherapy, the PROFILE 1007 trial, which compared crizotinib with pemetrexed or docetaxel as a second-line treatment in ALKi-naïve NSCLC pts, showed that the prevalence of BM was approximately 35 % in both arms, which is higher than that observed in the first-line PROFILE 1014 study (Table 1) [5]. Similar to this incidence, a retrospective exploratory analysis of the PROFILE 1015 and PROFILE 1007 trials showed that 275 (31 %) of the enrolled 888 *ALK*+ pts had BM without any symptoms [22]. A phase II study (AF-001JP) that investigated the efficacy of alectinib at a dose of 300 mg twice daily for ALKi-naïve NSCLC pts who had received one or more lines of chemotherapy showed almost the same prevalence (33 %) of BM as that seen in the retrospective study of PROFILE 1005 and PROFILE 1007 [7]. In the ASCEND-3 study of ceritinib in ALKi-naïve pts who had received one to three prior antineoplastic regimens, the prevalence of CNS metastasis was 40.3 % (50/124) [16].

**Table 1:** Frequency of BM in the trials regarding ALK inhibitors and efficacy of ALK inhibitors for BM in pts with *ALK*+ NSCLC

Trial	Treatment regimen	No. of pts	Frequency of BM, *n* (%)	Line of Tx	Prior ALKi	Target lesion	Intracranial response (target lesion)	
							DCR	RR
PROFILE 1007 [5]	Crizotinib	173	60 (35)	2nd	No	Not described	Not described	
	PEM or DOC	174	60 (34)					
PROFILE 1014 [6]	Crizotinib	172	45 (26)	1st	No	Not described	56 % at 24 weeks	Not described
	PEM + CBDCA or CDDP	171	47 (27)				25 % at 24 weeks	Not described
PROFILE 1005 and PROFILE 1007 [22]	Crizotinib	888	275 (31)	≥2nd	No	22 (previously untreated)	56 % at 12 weeks (previously untreated)	18 % (previously untreated)
AF-001JP [7]	Alectinib	46	15 (33)	≥2nd	No	Not described	Not described	
AF-002JG [8]	Alectinib	47	21 (45)	≥2nd	Yes (crizotinib)	9	77.8 %	55.6 %
NP28673 [19]	Alectinib	138	84 (61)	≥2nd	Yes (crizotinib)	35	85.7 %	57.1 %
NP28761 [20]	Alectinib	87	52 (60)	≥2nd	Yes (crizotinib)	16	100 %	68.8 %
JP28927 [53]	Alectinib	35	23 (65.7)	≥1st	Yes (29/35, crizotinib and other ALK inhibitors)	2	100 %	100 %
ASCEND-1 [17]	Ceritinib	246	124 (50.4)	≥1st	Yes (98/124, other ALK inhibitors)	29	58.6 %	34.5 %
ASCEND-2 [18]	Ceritinib	140	100 (71.4)	≥2nd	Yes (crizotinib)	33	84.8 %	39.4 %
ASCEND-3 [16]	Ceritinib	124	50 (40.3)	≥2nd	No	17	82.4 %	58.8 %
AP26113 [21]	AP26113	79	52 (66)	≥1st	Yes (71/79, crizotinib)	15	87 %	53 %
PF-06463922 [28]	PF-06463922	44^a^	52 (66)	≥1st	Yes (37/44, ALKi)	14	72 %	36 %

*BM* brain metastasis, *No*. number, *pts* patients, *Tx* therapy, *DCR* disease control rate, *RR* response rate, *IC* intracranial, *DOR* duration of response, *PEM* pemetrexed, *DOC* docetaxel, *CBDCA* carboplatin, *CDDP* cisplatin

^a^Thirty-three (75 %) and 11 (25 %) were *ALK*+ and *ROS1*+ patients

With regard to the incidence of BM in pts with *ALK* rearrangement previously treated with ALKi, several prospective studies have reported the detailed prevalence of BM in such populations, which ranges from approximately 40 to 70 %, as shown in Table 1 [8, 16–21, 28]. Almost all pts enrolled in these studies had been treated with crizotinib, suggesting that BM is very common in pts with crizotinib-treated *ALK*+ NSCLC. In fact, a retrospective study by Weickhardt et al. showed that 13 (46 %) among 28 cases of disease progression on crizotinib were identified to be due to BM [31]. Furthermore, a retrospective investigation of the PROFILE 1005 and PROFILE 1007 trials demonstrated that the CNS was the most frequently affected organ in cases of progressive disease (PD) in pts on crizotinib with or without a previous history of treatment for baseline BM, which ranged from 70 to 72 % [22]. In addition, with regard to the cumulative risk of IC metastasis in *ALK*+ NSCLC pts, Rangachari and colleagues reported that the incidence of BM in *ALK*-rearranged pts at 1, 2, and 3 years from the initial diagnosis was 23.8, 45.5, and 58.4 %, respectively [15]. Therefore, IC metastases are more commonly observed after the failure of antineoplastic agents including ALKi, which is one of the most confusing issues to be addressed in the management of pts with *ALK*-rearranged NSCLC.

## Antitumor activity of ALKi against BM in pts with *ALK*+ NSCLC

CNS metastasis is usually insensitive to chemotherapy, and the RR of chemotherapy for BM from solid tumors has been reported to be approximately 20 to 40 %, suggesting that the brain is a sanctuary site in the treatment of pts with advanced lung cancer [32]. With regard to the efficacy of targeted therapy for BM arising from lung cancer, several studies have reported that erlotinib, a representative targeted agent, might be effective for CNS lesions. Erlotinib has been found to achieve a longer survival time in *EGFR*-mutated pts with BM compared with gefitinib, and the higher efficacy of erlotinib versus gefitinib is assumed to be due to the higher concentration and penetration of erlotinib in the cerebrospinal fluid (CSF) [33, 34]. In addition, the proportion of CNS PD is higher in cases of gefitinib compared with erlotinib: gefitinib versus erlotinib, approximately 35 % versus up to 10 % [35–38]. Hence, the efficacy of molecular-targeted agents for BM has gradually been clarified.

With regard to the efficacy of ALKi for CNS metastasis in pts with *ALK* rearrangement-positive NSCLC, much evidence was reported at the ASCO Annual Meeting in 2015 (Table 1), and in this section, we review this evidence in detail. In order to clarify the antitumor effect of each ALKi agent, the DCR and RR for the target lesions are specified in Table 1.

### Crizotinib

The PROFILE trials demonstrated that crizotinib, a first-in-class ALK TKI, achieves a significantly higher response in the systemic lesions of *ALK*+ NSCLC pts as compared with chemotherapy; however, it was not fully clarified whether crizotinib is effective for CNS metastasis [3–6]. In the PROFILE 1014 trial, the IC DCR at 24 weeks after the start of treatment was reported and crizotinib and first-line standard chemotherapy of platinum agents with pemetrexed achieved rates of 56 and 25 %, respectively, with a statistically significant difference (Table 1). A large retrospective study of the PROFILE 1015 and PROFILE 1007 trials examined the efficacy of crizotinib for BM in pts with *ALK* rearrangement with or without a history of prior cranial radiotherapy [22]. Among 888 pts enrolled in these trials, 275 pts (31 %) were judged to harbor asymptomatic BM and divided into cases previously untreated or treated with radiotherapy (*n* = 109 and 166, respectively). The DCRs at 12 weeks in the previously untreated or treated BM cases were 56 % (95 % confidence interval (CI) 46–66) and 62 % (95 % CI 54–70), respectively, and the RRs of crizotinib in the target lesion in BM cases previously untreated or treated with radiation were 18 % (95 % CI 5–40) and 33 % (95 % CI 13–59), respectively. Although the difference in the RR observed between the two groups appears to be significant, attention should be paid to interpreting this result because radiotherapy for BM prior to enrollment in these studies might have affected the RR. The IC durations of response (DORs) in previously treated or untreated pts with a confirmed objective response was 26.4 (95 % CI 6.1–59.3) and not reached (95 % CI 6.0–59.9), respectively. Furthermore, the studies showed that systemic PFS was not affected by the presence or absence of BM at baseline. Intriguingly, non-target or new lesions in the brain accounted for 71 % of RECIST PD cases in pts with baseline BM and 25 % of PD cases in pts without baseline BM, suggesting that the CNS remained the dominant site of acquired resistance to crizotinib in the pts with or without BM at baseline.

Furthermore, several case reports have reported the contradictory efficacy of crizotinib for CNS lesions, including intramedullary spinal cord metastasis and leptomeningeal carcinomatosis, in *ALK*+ NSCLC pts [39–45]. The rapid and long-lasting response of BM to crizotinib was recently reported by two groups [39, 40], and high-dose crizotinib has been shown to temporarily control BM refractory to standard-dose crizotinib [42]. In contrast, some reports have suggested that BM is insensitive to crizotinib, which leads to the notion that the brain is a sanctuary site or Achilles heel [43, 44]. Importantly, a report by Costa and colleagues examined the concentration of crizotinib in the CSF of an *ALK*+ NSCLC pt who had progressed on crizotinib for BM and was treated with whole brain irradiation (WBI) and then resumed crizotinib after WBI [43]. That report demonstrated that concentration of crizotinib restarted after WBI in the CSF was as low as 0.616 ng/ml, while the plasma concentration of the agent was 237 ng/ml, with a CSF-to-plasma ratio of 0.0026. This result suggests that the possible poor BBB penetration of crizotinib might explain the failure of crizotinib to be effective for BM in cases of *ALK*+ NSCLC. Another report published by Metro et al. also analyzed the CSF concentrations of crizotinib in two pts with *ALK* rearrangement whose CNS lesions were successfully treated with crizotinib [46]. In one case, in which the pt was naïve to radiotherapy for CNS lesions and experienced a complete CNS response, the concentrations of crizotinib in the serum and CSF were 587 and 0.35 ng/ml, respectively, with a CSF-to-serum ratio of 0.0006. In another case, in which the pt progressed on crizotinib for BM and was treated with WBI and subsequently continued crizotinib with a temporary discontinuation during WBI, the concentrations of crizotinib in the serum and CSF were 800 and 0.80 ng/ml, respectively, with a CSF-to-serum ratio of 0.001. Intriguingly, despite the poor CNS concentration of crizotinib, the CNS lesions in these two pts were successfully tread with crizotinib, suggesting the possible involvement of other factors in the CNS accounting for the benefit of crizotinib. Based on these findings, a low CSF-to-serum ratio alone does not explain the insensitivity of crizotinib to BM, which should be clarified in further investigations.

### Alectinib

**Fig. 1:**
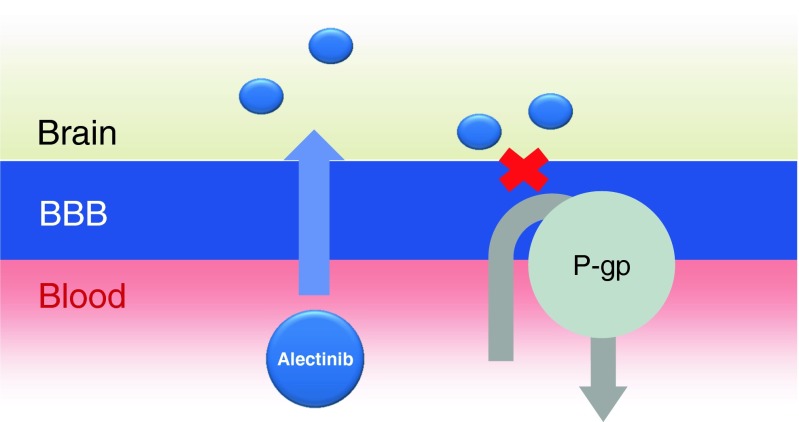
Alectinib is not a substrate of P-gp, which is expressed in the BBB and plays a key role in BBB penetration. *BBB* blood-brain barrier, *P-gp P*-glycoprotein

Alectinib/CH5424802 was developed to be highly selective against ALK and later identified to block ret proto-oncogene [47, 48]. Preclinical experiments have demonstrated that this agent is capable of blocking mutated forms of ALK, including a gatekeeper mutation of L1196M conferring crizotinib resistance, as well as wild-type ALK [47, 49]. With regard to the antitumor efficacy of alectinib for BM, intracranial tumor implantation mouse models of *ALK*+ NSCLC cells have demonstrated that alectinib potently induces IC tumor regression compared with crizotinib, resulting in prolonged survival [26, 27]. This potent antineoplastic activity of alectinib against BM is assumed to be due to high rate of penetration of the agent into the brain, and intriguingly, it has been suggested that alectinib might not be transported by *P*-gp, which is expressed in the BBB and plays a key role in BBB penetration (Fig. 1) [26, 50]. In addition, data for five alectinib-treated pts with available plasma and CSF samples showed a relationship between the concentration of alectinib in the CSF and paired systemic unbound alectinib [8].

The phase II portion of the AF-001JP trial included 33 % pts with BM among 46 pts enrolled at baseline, although the detailed responses have not yet been reported [7, 51]. Four (33 %) of 12 pts who experienced PD showed CNS progression, and half of the pts with baseline IC metastasis continued to be treated with alectinib without any PD at brain or systemic sites. The median PFS, which was not reached and was estimated to reach beyond 29 months, was affected by the presence of BM at baseline, i.e., 35.3 months versus not reached in the pts with or without BM at baseline, respectively. Furthermore, other studies investigating alectinib, such as AF-002JG, NP2867, and NP28761, were recently designed to evaluate crizotinib-resistant or intolerant pts with *ALK* translocation and showed detailed data regarding the efficacy of alectinib for IC lesions [8, 19, 20]. In the AF-002JG study, among 21 pts with BM at baseline, six (29 %), five (24 %), eight (38 %), and two (10 %) achieved a complete response (CR), partial response, stable disease, and PD, respectively [8]. When limited to nine pts with measurable BM lesions, the objective RR (ORR) and DCR were as high as 55.6 and 77.8 %, respectively. Intriguingly, one of two pts experiencing IC PD, despite a systemic good response, had been treated with stereotactic radiotherapy for BM, and only one lesion was enlarged, with other lesions being decreased. Thereafter, surgical resection was performed for the enlarging lesion, which was confirmed to be necrotic, without any viable cancer cells, suggesting pseudo-progression (PsP). In addition to this report, Ou et al. reported two pts who experienced radiation necrosis presenting as PsP when treated with alectinib for previously irradiated BM [52]. These results underline the importance of distinguishing truly progressive disease from PsP when treating previously radiated IC lesions with alectinib, although the optimal way to do this has yet to be established, other than surgical resection.

The phase II NP28673 and NP28761 studies are aimed to examine the efficacy and safety of alectinib at a dose of 600 mg twice daily for pts with *ALK*+ NSCLC who progressed on or did not respond to crizotinib [19, 20]. In the NP28783 trial, the frequency of BM was 84 (61 %) among 138 pts and 35 pts had measurable CNS lesions. The IC ORR and DCR in pts with measurable BM were 57.1 % (95 % CI 39.4–73.7) and 85.7 % (95 % CI 69.7–95.2), respectively, and the CR was observed in seven pts (20.0 %). In addition, the DOR in the CNS was as long as 10.3 months (95 % CI 7.6–11.2). Moreover, the RR in the pts treated with or without prior radiation was 39.3 and 52.2 %, respectively, and considering the possibility of PsP, attention should be paid to interpreting the slightly low RR values noted in pts previously treated with radiation. The NP28761 trial also demonstrated the excellent efficacy of alectinib for IC lesions: the RR and DCR in the pts with measurable BM at baseline were 68.8 and 100 %, respectively. The antitumor activity of alectinib for BM in cases of *ALK*+ NSCLC pre-treated with crizotinib was also reported in the JP28927 study [53]. With regard to the relapse pattern, nine out of 23 (65.7 %) pts with baseline BM were identified to progress on alectinib and all pts, except one, progressed at sites other than the brain. Furthermore, alectinib has been reported to be effective for CNS metastasis associated with leptomeningeal involvement previously treated with crizotinib and alectinib [25]. Therefore, alectinib is highly effective for CNS lesions in *ALK*+ NSCLC pts. Difference in systemic and IC efficacy between 300 and 600 mg twice daily, if any, should be investigated in future studies.

### Ceritinib

Ceritinib (LDK378), a highly selective, orally available ALK inhibitor, has been shown to inhibit ALK with 20-fold potency to that of crizotinib and is effective for ALK with several mutations that confer resistance to crizotinib in preclinical experiments [54]. In humans, a phase I study investigating ceritinib demonstrated ORR in pts with or without prior crizotinib treatment of 56 and of 58 %, respectively [9], and crizotinib followed by ceritinib has been shown to prolong combined PFS [55]. With regard to the efficacy of ceritinib for IC metastasis, a preclinical rat model showed that ceritinib penetrates the BBB with a brain-to-blood exposure ratio of approximately 15 % [56], and the IC efficacy of ceritinib has been observed in pts previously treated with or without crizotinib [9].

More detailed data on the efficacy of ceritinib for CNS metastasis were reported in the ASCEND studies. The ASCEND-1 study included 124 (50.4 %) pts with baseline BM among 246 enrolled pts, and 98 of the pts with BM had been treated with other ALK inhibitors agents. The IC RR and DCR in the pts with measurable BM were 34.5 % (95 % CI 17.9–54.3) and 58.6 % (95 % CI 38.9–76.5), respectively [17]. Prior ALKi treatment affected the RR and IC DCR: the RR and IC DCR values in the pts with or without prior ALKi treatment were 29.2 and 60.0 %, and 58.3 and 60.0 %, respectively. The IC median PFS was reported to be 8.3 and 7.0 months and not estimable in all pts, the pts with prior ALKi and the ALKi-naïve pts. The ASCEND-2 trial is a phase II study of ceritinib in pts with *ALK*+ NSCLC previously treated with chemotherapy and crizotinib, and all pts had a history of crizotinib treatment as the last prior systemic anticancer therapy [18]. One hundred pts (71.4 %) had a history of prior radiotherapy for BM, and the IC RR and DCR in 33 pts with target lesions in the brain were 39.4 % (95 % CI 22.9–57.9) and 84.8 % (95 % CI 68.1–94.9), respectively. In addition, the ASCEND-3 study aimed to investigate the antitumor efficacy and safety of ceritinib in ALKi-naïve pts with *ALK* rearrangement who had progressed on one to three lines of chemotherapy [16]. Among 50 (40.3 %) pts with BM at baseline, 27 pts (54.0 %) had been treated with prior radiotherapy to the brain. The IC RR and DCR in 17 pts with target lesions in the brain were 58.9 % (95 % CI 32.9–81.6) and 82.4 % (95 % CI 56.6–96.2), respectively. These findings demonstrate that ceritinib is highly effective for pts with BM, and intriguingly, the antineoplastic effect of ceritinib against BM is high for pts without a history of ALKi treatment compared with those pre-treated with other ALK inhibitors.

### Other ALK inhibitors

Other ALK inhibitors, such as AP26133 and PF-06463922, have been also shown to be effective for IC metastasis [21, 28]. The phase I/II study of AP26113 included 137 pts with advanced malignancies, including 79 pts with *ALK*-rearranged NSCLC, of whom 52 (66 %) of 79 *ALK*+ NSCLC pts had BM at baseline [21]. The IC ORR and DCR in the *ALK*+ pts with measurable BM were as high as 53 and 87 %, respectively. With regard to PF-06463922, the IC RR and DCR were 36 and 72 %, respectively [28], and the high IC efficacy of the agent was confirmed in preclinical IC models [57]. Furthermore, IC data for other novel ALK inhibitors, such as X-396, TSR-11, and so on, are anticipated to be reported in the near future [58, 59].

## Beyond PD use of ALKi for BM

In clinical settings using molecular-targeted agents, physicians usually experience cases of PD only in the CNS [60]. Several retrospective studies have demonstrated that the use of crizotinib beyond progressive disease (CBPD) combined with cranial radiotherapy for CNS PD might offer a survival benefit [31, 61, 62]. Takeda and colleagues retrospectively investigated seven pts with *ALK* rearrangement who progressed on crizotinib in isolated CNS lesions and resumed crizotinib after local ablative therapy (LAT), including stereotactic radiotherapy (SRT) for three pts and WBI for four pts [60]. The median PFS from the first initiation of crizotinib was 5.5 months, and all pts were able to continue the treatment with crizotinib for at least 4 months after LAT without disease progression, suggesting that the addition of LAT to crizotinib for isolated BM might prolong the total PFS. In addition to this study, Weickhardt et al. examined LAT for oligo-progressive disease, including brain and extra-CNS lesions, in 25 pts with *EGFR*-mutated or *ALK*-rearranged NSCLC who progressed on erlotinib or crizotinib and were judged to be suitable for LAT [31]. The PFS achieved with erlotinib and crizotinib was 13.8 and 9.0 months, respectively, and LAT for CNS, which was a site of first progression, prolonged the PFS for more than 7.0 months. Although grade 3/4 fatigue was reported in two pts who received WBI, no other grade 3/4 radiation-related toxicities were observed. Furthermore, a retrospective investigation of *ALK*+ pts enrolled in the PROFILE 1001 or PROFILE 1005 trials who were able to continue CBPD demonstrated that CBPD might be associated with prolonged survival: the OS from the time of PD in the pts with or without CBPD was 16.4 versus 2.9 months, respectively (hazard ratio (HR) 0.27, 95 % CI 0.17–0.42; *P* < 0.0001) [61]. In addition, the OS from the first dose of crizotinib in the pts with or without CBPD was 29.6 and 10.8 months, respectively (HR 0.30, 95 % CI 0.19–0.46; *P* < 0.0001). It should be noted that 51 % of the pts with CBPD had the brain as a site of PD, while the pts without CBPD had both the liver (37 %) and the brain (28 %) as PD sites, suggesting that pts with progressive disease in the CNS might benefit significantly from CBPD.

Therefore, CBPD for CNS PD appears to prolong survival. Hence, should physicians always consider CBPD in pts who relapse on crizotinib in isolated CNS lesions? If we have a choice to use next-generation ALKi, such as alectinib, after failure of or intolerance to crizotinib, the answer seems to be *no*. As described above, next-generation ALK inhibitors, specifically alectinib, are highly effective for CNS lesions pre-treated with crizotinib. In addition to this point, considering the possible long survival achieved with two or more ALK inhibitors and cytotoxic chemotherapy and the potential for late complications of radiotherapy, such as radiation necrosis and encephalopathy, even isolated CNS metastases should be considered truly *progressive* and crizotinib should be changed to another ALKi. However, if all available ALK inhibitors are completed, ALKi beyond PD combined with LAT for BM should be considered, because cytotoxic chemotherapy is usually ineffective for such lesions. Since these strategies are not based on definitive data, future studies are warranted to investigate which strategy, using ALKi beyond PD concurrently with LAT for BM or switching to other ALK inhibitors or cytotoxic chemotherapy, is better in terms of the survival benefit and safety.

## Conclusion

We herein described the detailed data on the frequency of BM and efficacy of various ALK inhibitors for BM. Although the brain is thought to be one of the most invulnerable sites, recent trials have demonstrated that novel ALK inhibitors exhibit high antineoplastic effects on CNS lesions. Future studies are warranted to elucidate the most optimal management of BM in pts with *ALK*-rearranged NSCLC.
